# Silencing Calumenin Expression via Artificial MicroRNA, a Potential Breakthrough for Inhibiting Proliferation, Halting Migration, and Triggering Apoptosis in Breast Cancer Cells

**DOI:** 10.34172/apb.025.43819

**Published:** 2025-06-11

**Authors:** Zahra Amiri, Fatemeh Bahrami, Babak Jahangiri, Arash Javeri, Frouzandeh Mahjoubi, Nahid Nafissi, Mohammad Zaefizadeh, Fatemeh Masoumi, Alireza Zomorodipour

**Affiliations:** ^1^Department of Molecular Medicine, Institute of Medical Genetics, National Institute of Genetic Engineering and Biotechnology, Tehran, Iran; ^2^Department of Stem Cell and Regenerative Medicine, Institute of Medical Genetics, National Institute of Genetic Engineering and Biotechnology, Tehran, Iran; ^3^Department of Medical Genetics, Institute of Medical Genetics, National Institute of Genetic Engineering and Biotechnology, Tehran, Iran; ^4^Hazrat-e Rasool General Hospital, Iran University of Medical Science, Tehran, Iran; ^5^Department of Biology, School of Sciences, Ardabil branch, Islamic Azad University, Ardabil, Iran

**Keywords:** Apoptosis, Artificial microRNA, Breast cancer, Calumenin, Cell proliferation, Metastasis

## Abstract

**Purpose:**

Calumenin (CALU) is a calcium-binding protein involved in several physiological processes, exhibiting tumor-specific expression variation and emerging as a potential player in cancer progression. This study aimed to investigate the correlation between CALU and clinicopathological features in breast cancer (BC) and perform a functional assessment of CALU based on a microRNA-mediated knockdown approach.

**Methods:**

The BC tissues’ CALU expression was measured by q-RT-PCR. We looked at correlations between changes in CALU expression and clinicopathological characteristics. We adopted a CALU knockdown approach using an artificial microRNA (amiR), expressed through an episomal vector, in BC cell lines. Epithelial to mesenchymal transition (EMT) markers were then assessed, and cell cycle, migration, proliferation, and apoptosis were analyzed.

**Results:**

When compared to the normal surrounding tissues, the BC tissues showed a 3.4-fold increase in CALU expression. This was significantly correlated with clinicopathological parameters such as histological grade, Ki-67 expression, TNM stage, lymph node involvement, and vascular lymph invasion. Key EMT markers, including GSC, MMP2, TIMP1, TGF1, SLUG, ZEB1, ZEB2, SNALI1, and TWIST1, were downregulated as a result of CALU knockdown, which prevented cell migration and proliferation and caused cell cycle arrest and apoptosis in the BC cell lines.

**Conclusion:**

The results of the amiR-mediated knockdown approach support the findings that CALU is a potential promoter of BC, as evidenced by the upregulation of CALU in BC tissues and its correlation with clinicopathological features, which highlights its role in BC progression.

## Introduction

 Calumenin (CALU) is a multiple EF-hand calcium (Ca)-binding protein and a member of the Cab45/reticulocalbin/ERC-45/CALU (CREC) protein family. The mammalian CREC family members, mainly located in various secretory pathway compartments, are characterized by multiple EF-hands and participate in different physiological processes, and Ca homeostasis is the primary outcome of their functions.^1,2^

 Cancer is a complex and multifaceted disease characterized by uncontrolled cell growth, evasion of apoptosis, invasion of surrounding tissues, and metastasis to distant organs.^3^ It is imperative to comprehend the molecular mechanisms that propel cancer progression to design potent and efficacious treatment.^4^ Emerging evidence in recent years suggests that CALU may play a significant role in cancer promotion, and its association with more malignant phenotypes and shorter survival rates for patients has been widely reported.^5,6^

 Metastasis is a primary cause of the majority of cancermorbidities and mortalities, and the development of effective treatment programs requires a better understanding of the molecular mechanisms behind this process.^7^ CALU has been suggested to promote metastasis of cancer cells by interacting with extracellular matrix (ECM) components, such as fibronectin and laminin.^8^ CALU also has emerged as a regulator of epithelial to mesenchymal transition (EMT), promoting the transition from an epithelial to a mesenchymal phenotype. This Ca-binding protein enhances the expression of EMT-inducing transcription factors, such as Snail, Twist, and ZEB1, while downregulating epithelial markers like E-cadherin and promoting cancer cell migration, invasion, and resistance to therapy.^5,6^ Furthermore, CALU may inhibit angiogenesis, a critical process for tumor growth and metastasis through fibulin-1.^9,10^

 The human *CALU* gene produces 15 isoforms, among them CALU 1–14, with N-terminal signal peptides, are either localized in secretory pathway compartments or secreted into the extracellular space, and CALU-15 shuttles between nucleus and cytoplasm, due to lack of signal peptide.^8^ Among the CALU isoforms, CALU-15 is reportedly involved in cell migration by promoting the formation of ﬁlopodia, an actin-rich ﬁnger-like membrane protrusion with a key role in metastasis.^8^ CALU-15, a nuclear-localized and phosphorylation-dependent CALU isoform, promotes filopodia formation and cell migration by upregulating growth differentiation factor-15 (GDF-15), a transforming growth factor-beta (TGF-b)-like cytokine.^8^ GDF-15 has been shown to have both antitumor and antiproliferative properties, although this remains a highly controversial topic.^11-14^ Due to its pleiotropic effects, GDF-15 operates under the tight control of many regulatory pathways and participates in a variety of cellular processes.^15-20^ Consequently, CALU-15 was suggested as a new regulator of GDF-15.^8^

 In contrast, the extracellular CALU isoforms suppressthe signal-regulated kinase 1 and 2 (ERK1/2) signaling and inhibit cell migration.^21^ These pieces of evidence suggest the potential prognostic and therapeutic values of CALU isoforms in cancers. In our previous study, we introduced a panel of CALU/ARUKA/MCM2 genes with high accuracy in discriminating the biopsies of colon and lung cancers from healthy samples, where the high expression levels of these genes are negatively correlated with the patient’s survival rate.^22^

 The widespread involvement of microRNAs (miRNA) in many human diseases, including various cancers, makes them attractive tools for targeted therapeutic strategies.^23^ In this regard, synthetic miRNAs, by silencing the gene(s) of interest, have shown great promise as a new class of targeted therapeutic agents for treating various human diseases, including cancer.^24^ The most attractive aspect of miRNA therapeutics is its ability to target almost any gene that may not be possible with small molecules or protein-based drugs, although there are still challenges regarding their application at the clinical level.^25^

 Although there is a wealth of evidence supporting the critical role of CALU in many cellular and cancer-related processes, little is known about its function in breast cancer (BC).^26^ In this study, we aimed to investigate the possible relationship between CALU expression patterns and clinicopathological and demographic characteristics among women with BC. Besides, using a CALU-specific artificial miRNA (amiR), we have adopted a miRNA-mediated knockdown strategy in two BC cell lines to address the CALU function in BC and its association with proliferation, apoptosis, and metastasis potential.

## Materials and Methods

###  Human samples collection and cell culture

 The National Institute of Genetic Engineering and Biotechnology (NIGEB) of Iran’s ethics committee accepted this work under ethics approval codes IR.NIGEB.EC.1402.11.29.D and IR.NIGEB.EC.1402.11.29.E. This study included 55 BC women who had received BC surgery at Khatam-Ol-Anbia Hospital in Tehran, Iran. Fresh tissue specimens (tumor tissues and their normal adjacent tissues) were collected in separate sterile tubes, frozen, and stored at -70°C. An expert pathologist confirmed a histologic diagnosis for all samples. Informed written consent was obtained from patients for all tissue samples by the clinicians.

 The SK-BR-3 and MDA-MB-231 BC cell lines were purchased from the Iranian Biological Resource Center (Tehran, Iran) and cultured in high-glucose DMEM supplemented with 10% heat-inactivated fetal bovine serum (FBS) (Gibco, USA), 100 U. mL^-1^ penicillin, and 100 μg. mL^-1^ streptomycin (Sigma, USA) at 37 °C in a 5% CO2-humidified incubator. After the BC cell lines had reached a 70%-80% confluent monolayer, they were passaged and used for subsequent experiments.

 The cloning processes were carried out using the E. Coli strain DH5α (Stratagene, USA). For PCR, PCR cloning, and sub-clonings, MWG-Germany’s produced primers were employed (Table 1). Thermo Fisher Scientific, USA, provided the enzymes XbaI, XhoI, HindIII, StuI, PstI, T4 DNA ligase, DNase, and reverse transcriptase (M-MuLV).

**Table 1:** Primers used in this study

**Gene name**	**Accession **	**Forward primer Seq. (5’-3’)**	**Forward primer location**	**Reverse primer Seq. (5’-3’)**	**Reverse primer location**	**PCR product size (bp)**
ACTB	NM_001101.5	GAGACCTTCAACACCCCAGCC	Exon 4	AGACGCAGGATGGCATGGG	Exon 4	161
CALU	NM_001219.5	GATGGTTAGAGATGAGCGGAG	Exon 4	ATCTTCCATTGTTTCCTGTACTACT	Exons 4-5 boundary	142
SNAI1	NM_005985.4	ATGCACATCCGAAGCCACAC	Exon 2	CACTGGTACTTCTTGACATCTG	Exon 3	203
SNAI2	NM_003068.5	GCGATGCCCAGTCTAGAAAAT	Exon 2	ACCTGTCTGCAAATGCTCTGTT	Exon 3	225
TIMP1	NM_003254.3	TCCTGTTGTTGCTGTGGCTGA	Exon 2	CCTTTATACATCTTGGTCATCTTG	Exons 3-4 boundary	181
MMP2	NM_004530.6	TTGGCTACACACCTGATCTGG	Exon 3	GAGTCCGTCCTTACCGTCAAA	Exon 4	185
GSC	NM_173849.3	GAAAGTGGAGGTCTGGTTTAA	Exons 2-3 boundary	TACCTTCCTCTTCCCTCTTCT	Exon 3	143
TWIST1	NM_000474.4	TACATCGACTTCCTCTACCAG	Exon 1	GGAAACAATGACATCTAGGTCTC	Exons 1-2 boundary	203
ZEB1	NM_001128128.3	CAGATGATGAAGACAAACTGCATA	Exon 2	CCCTTCCTTTCCTGTGTCAT	Exons 2-3 boundary	176
ZEB2	NM_014795.4	GGACAGATCAGCACCAAATG	Exons 6-7 boundary	ATGTGCGAACTGTAGGAACCA	Exon 8	190
TGFB1	NM_000660.7	CTATTGCTTCAGCTCCACGGA	Exons 5-6 boundary	AGGACCTTGCTGTACTGCGT	Exons 6-7 boundary	171
GDF-15	NM_004864.4	AGAAGTGCGGCTGGGATCC	Exons 1-2 boundary	TCTTGCAAGGCTGAGCTGAC	Exon 2	180
GAPDH	NM_001289746.2	AAGGTGAAGGTCGGAGTCAAC	Exon 2	GGGGTCATTGATGGCAACAATA	Exon 3	102

###  Plasmids 

 Construction of the CALU-specific amiR-6 was described previously.^27^ In summary, sequence comparison of fifteen CALU transcript isoforms led to the identification of a conserved sequence (GGAAACAATGGAAGATATA) that served as a template for the design of amiR-6 capable of targeting both nuclear and non-nuclear CALU variants.

**Figure 1:**
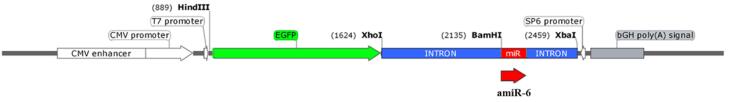
A schematic view of EGFP-Int-amiR-6 expression cassette, inserted within the pcDNA3 plasmid. The CALU-specific miRNA (amiR-6, Red), within a truncated FIX intron 1 (Blue), downstream to an EGFP coding sequence (Green) are shown

 A previously constructed CALU-specific amiR expressing plasmid, namely FIX-Int-miR6, expressing amiR-6,^27^ was modified by replacing the FIX cDNA with a 747 bp EGFP-coding DNA to end up with plasmid EGFP-Int-miRNA-6 (amiR-6). The forward and reverse primers used for PCR amplification of EGFP were designed to contain a mammalian Kozak sequence (GCCRCCATGG)^28^ in the start codon context and a TAA codon as a stop codon, which ensured higher translational efficiency. In the constructed plasmid (EGFP-Int-miRNA-6), the CALU-specific amiR-6 coding sequence was inserted in the EGFP 5’-UTR (Figure 1). A miRNA-less EGFP expressing plasmid was constructed and used as a negative control (mock). Restriction analysis and nucleotide sequence analysis were used to verify the recombinant plasmids. A comparison of nucleotide sequences against the gene bank was done using BLAST.^29^

###  Real-time quantitative PCR (RT-qPCR)

 Total RNA was isolated from tissues and cells using a High Pure RNA Isolation Kit (Roche, Germany). A NanoDrop spectrophotometer was used to measure RNA concentrations (Thermo Fisher, USA). PrimeScript^TM^ 1st Strand cDNA Synthesis Kit (Takara Bio, Japan) was applied to perform reverse transcription on cDNA. The concentration and purity of the extracted cDNA were estimated using a NanoDrop Spectrophotometer (Thermo Scientific, USA). PCR was used to assess the quality of the generated cDNA using GAPDH-specific primers. The levels of expressed amiR-6 and mRNAs were compared to the levels of endogenous U6 and GAPDH, respectively, to normalize their expression. The 2^-ΔΔCt^ method was used for evaluating the real-time PCR results.^30^ The assays were carried out in triplicates.

###  Cell transfection

 The BC cell lines, plated in individual wells of 6-well plates, underwent transfection with amiR-6 or mock through Lipofectamine 2000 (Invitrogen), as per the manufacturer’s guidelines. After 48h, the cells were harvested, and the later assays were performed.

###  Cell proliferation assay

 The amiR-6 effect on the proliferation of BC cell lines was evaluated through a trypan blue dye viability assay or an exclusion test. Before the trypan blue exclusion test, a 24-well plate was seeded with 8 × 10^4^ SK-BR-3 or MDA-MB-231 cells. Afterward, the cells underwent a transfection process involving the introduction of either amiR-6 or mock plasmid. Following 48 hours post-transfection, the number of living BC cells was determined through hemocytometer counting.

###  Cell cycle distribution analysis

 For the cell cycle analysis of the transfected BC cell lines after 48 hours, a total of 1 × 10^6^ cells per group were incubated with RNase A (10 mg. mL^-1^) for 1 h at 37 °C and stained with propidium iodide (PI) (50 µg. mL^-1^) in dark. The stained cells were analyzed with a BD FACSCalibur flow cytometer and FlowJo software (version 7.6.1). To ensure reproducibility, the experiment was repeated four times.

###  Evaluation of apoptosis 

 Cell apoptosis was detected by flow cytometry analysis.^31^ Briefly, the BC cell lines were seeded onto 6-well plates and transfected with amiR-6, or mock. The transfected BC cell lines were rinsed with phosphate-buffered saline (PBS) and re-suspended in 100 µL binding buffer at 48 h post-transfection. A two-step staining was performed on the cells, once with 5 µL of Annexin V-FITC (1 µg.mL^-1^) for 10 minutes and once with 5 µL of PI (1 µg.mL^-1^) for 5 minutes at room temperature, and in the dark. In the last step, FlowJo software (version 7.6.1) and a BD FACSCalibur flow cytometer were used to evaluate the apoptotic cells.

 Acridine orange/ethidium bromide (AO/EB) staining was used to detect apoptotic cells. The BC cells were incubated in 6 well plates up to 80% confluency before transfection with amiR-6 or mock and incubation at 37 °C for 48 h. The cells were washed twice with PBS before being exposed to a 1:1 combination of acridine orange and ethidium bromide (Sigma-Aldrich, USA) solution (100 µg.mL^-1^ each). Using an inverted fluorescent microscope, the cells were instantly analyzed.

###  Wound healing assay

 To perform a wound-healing assay, the transfected cells were grown in four-well tissue culture plates to 80% confluency. Using a sterile pipette tip, a gentle scrape was made to create a small linear scratch in the confluent monolayer before being washed with PBS twice to remove the detached cells and incubated in a low serum medium (1% FBS) for 72 h at 37 °C in 5% CO2 and imaged every 24 h, using an inverted microscope and analyzed by image analysis software (ImageJ, National Institute of Health, Bethesda, MD, USA). The wound healing degree was estimated by the distance passed by cells into the scratched area. The presented data is an average of three independent experiments.

###  Transwell migration assay

 To investigate the amiR-6 impact on migration, a transwell assay was carried out on the transfected BC cells using transwell plates (SPL) with an 8.0 µm pore size. Briefly, 1 × 10^5^ MDA-MB-231 and SK-BR-3 cells were transfected with amiR-6. After 48 h, the cells were trypsinized, rinsed with PBS, added to a serum-free medium, and cultured in the upper chamber of the inserts. As a chemoattractant, 10% FBS medium was added to the lower chamber. After culturing with 5% CO2 at 37 °C for 48 h, cotton wool was used to gently remove the cells that did not migrate through the pores. The inserts were then observed by inverted microscopy after being fixed with 20% methanol, stained with 0.2% crystal violet, and dried. Five distinct fields were used to count the migrated cells.

###  Statistical analysis

 The normal distribution of data was confirmed using SPSS version 22 software and the Kolmogorov-Smirnov test.^32^ The relative gene expression was obtained using the Pfaffl method.^33^ To investigate the possible relationship between the *CALU* transcription and clinicopathological features, SPSS version 22 software, independent-sample t-test, one-way ANOVA, and Duncan’s multiple range test were used. A 95% confidence interval was considered for all quantitative tests, and *P* values less than 0.05 were considered significant.

## Results and Discussions

###  Evaluation of relative CALU mRNA expression in tumor vs. normal breast tissues

**Figure 2:**
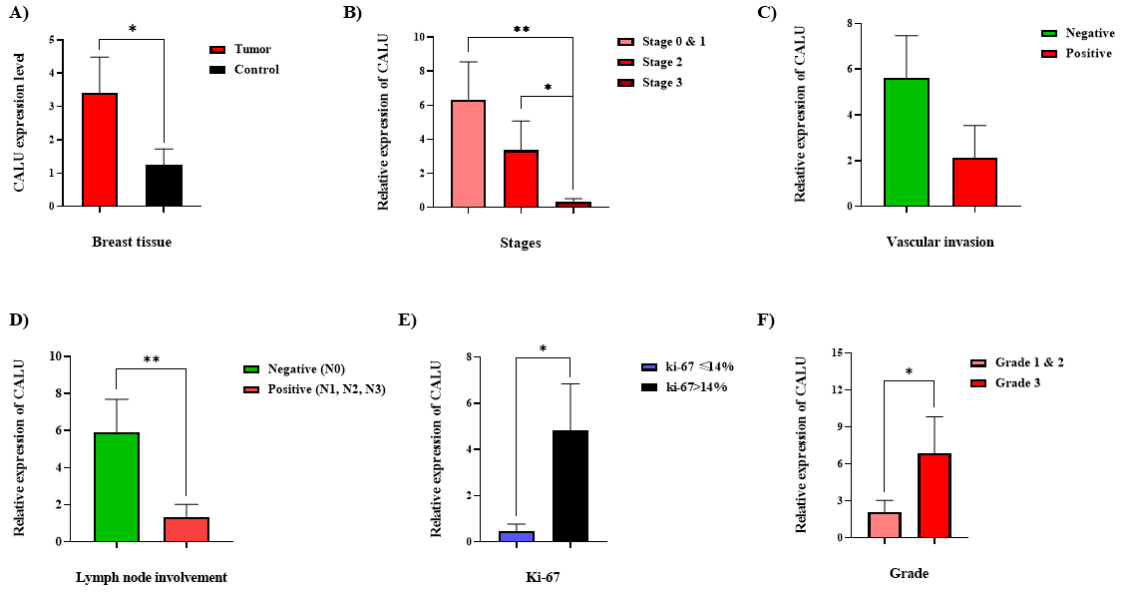
The CALU transcription assessment, concerning clinicopathological features of the BC patients. (A) Comparison of the CALU transcription in tumor and normal tissues in women with BC. The CALU transcriptions at different TNM stages of BC (B), in breast tumor samples with the negative status of vascular invasion compared with positive status (C), in tumor samples from patients with negative status of lymph node involvement compared with those of samples with positive status (D), at different levels of ki-67 (E), and different histological grades among BC tissues (F). The use of asterisks denotes notable variances in comparison to the control group. * = *P* ≤ 0.05, ** = *P* ≤ 0.01

 Evaluation of CALU expression among BC patients, based on the results obtained from RT-qPCR, showed a 3.4-fold increase in CALU transcript levels in breast tumor tissues compared to normal adjacent tissues (Figure 2A). Our data aligns with findings from Pan-cancer analyses, which reveal that CALU is consistently upregulated in BC, particularly the aggressive basal-like (triple-negative) subtype, and associated with poor prognoses.^26^ In vitro studies further demonstrate high CALU expression in triple-negative BC (TNBC) reverses EMT and inhibits migration, suggesting its significant role in promoting the mesenchymal transition and aggressive potential of this subtype.^26^ While the specific mechanisms and relationship to hormone receptor status warrant further investigation, CALU holds promise as a diagnostic or prognostic marker, especially in TNBC. Further investigation of CALU expression in Luminal A/B and HER2-enriched subtypes35 could also reveal valuable insights into its role in BC progression.

 Other studies also revealed a 10-fold increase in CALU expression in breast tumor interstitial fluid,^34^ and a 4-fold increase in CALU expression in a metastatic breast cell line compared to non-metastatic cells.^35^ Variation in the CALU expression during tumor progression was reported in other cancers, although the variation pattern is tissue-specific.^1^ In most cancer types, such as oral cancer,^36^ colon and lung cancers,^22^ and more malignant gliomas,^5^ enhancement of the CALU expression has been evidenced. In contrast, down-regulation of CALU in metastatic cell lines of head and neck,^37,38^ hepatocellular and pancreatic carcinoma,^2,21^ and lung squamous cell carcinoma,^39^ were shown. Differential regulation of the CALU has been observed in various malignancies, indicating its potential as a prognostic marker.^40^

###  Correlations between clinicopathological features and CALU expression

 Classification of the BC tissue samples was performed based on various clinicopathological features such as pathological type, histological grade, Ki-67 status, tumor size, lymph node involvement, TNM (stands for tumor size, lymph node number and position, and metastasis) staging, receptor status, including estrogen receptor (ER), progesterone receptor (PR), and human epidermal growth factor receptor 2 (HER-2), in addition to features such as necrosis, calcification, vascular invasion, and perineural invasion, outlined in Table 2. The analysis of the CALU expression variations, considering the clinicopathological features, demonstrated significant correlations between the CALU expression and features, including TNM staging, lymph node involvement, vascular invasion, histological grading, and Ki-67 index (Figure 2).

**Table 2:** Correlations between the BC clinicopathological features and CALU expression.

**Clinicopathological features**	**Different states**	**The number of patients (%)**	**CALU expression (mean±SEM)**	***P*** ** value**
Age (year)	˂45	15 (27.27)	3.28 ± 1.59	0.79
	45-55	22 (40)	4.56 ± 2.18	
	˃55	16 (29.09)	2.34 ± 1.49	
	Not known	2 (3.63)	NA	
Pathological type	IDC	35 (63.63)	3.67 ± 1.39	0.82
	ILC	9 (16.36)	3.74 ± 2.81	
	Others	8 (14.54)	3.11 ± 2.73	
	Not known	3 (5.45)	NA	
Histological grade	1 & 2	37 (67.27)	2.13 ± 0.90	0.03
	3	16 (29.09)	6.76 ± 2.89	
	Not known	2 (3.63)	NA	
Ki-67	≤ 14%	7 (12.72)	0.55 ± 0.41	0.05
	˃14%	26 (47.27)	4.85 ± 1.99	
	Not known	22 (40)	NA	
Tumor size	≤ 2	22 (40)	4.76 ± 1.80	0.39
	2-5	28 (50.90)	2.86 ± 1.52	
	˃ 5	2 (3.63)	0.99 ± 0.80	
	Not known	3 (5.45)	NA	
Lymph node Involvement	Negative	26 (47.27)	5.80 ± 2.02	0.005
	Positive	27 (49.09)	1.34 ± 0.77	
	Not known	2 (3.63)	NA	
TNM stage	0 & 1	16 (29.09)	6.37 ± 2.36	0.02
	2	24 (43.63)	3.37 ± 1.76	
	3	12 (21.81)	0.34 ± 0.16	
	Not known	3 (5.45)	NA	
ER	Positive	41 (74.54)	4.34 ± 1.40	0.56
	Negative	11 (20)	0.81 ± 0.32	
	Not known	3 (5.45)	NA	
PR	Positive	39 (70.90)	3.92 ± 1.39	0.83
	Negative	13 (23.63)	2.60 ± 1.68	
	Not known	3 (5.45)	NA	
HER-2	Positive	12 (21.81)	4.10 ± 2.36	0.99
	Negative	26 (47.27)	2.02 ± 0.97	
	Not known	17 (30.90)	NA	
Necrosis	Present	25 (45.45)	3.25 ± 1.69	0.62
	Absent	21 (38.18)	3.79 ± 1.68	
	Not known	9 (16.36)	NA	
Calcification	Present	9 (16.36)	2.97 ± 2.20	0.84
	Absent	38 (69.09)	4.20 ± 1.44	
	Not known	8 (14.54)	NA	
Vascular invasion	Present	27 (49.09)	2.12 ± 1.42	0.01
	Absent	23 (41.81)	5.64 ± 1.84	
	Not known	5 (9.09)	NA	
Perineurial invasion	Present	11 (20)	3.11 ± 2.32	0.60
	Absent	34 (61.81)	3.72 ± 1.43	
	Not known	10 (18.18)	NA	

 The samples were categorized into groups 1, 2, and 3, which correspond to stages 1/0, 2, and 3, respectively, based on the TNM staging (Figure 2B). According to an intergroup analysis, samples from stages 1.0 and 3 had the highest and lowest CALU transcription levels, respectively, with a significant difference (P-value < 0.02). Patients were classified as either positive or negative for vascular invasion depending on whether the disease recurred or did not. Patients with negative vascular invasion had higher levels of CALU expression than those with positive vascular invasion; however, this difference was not statistically significant (*P* value = 0.052) (Figure 2C). Patients’ status was classified as either positive or negative based on the involvement of lymph nodes. Compared to individuals with positive status, those with negative status had noticeably greater amounts of CALU transcripts (Figure 2D).

 The analyses revealed that in BC tissues, the highest amount of CALU transcription happened between stages 0 and 1 when vascular invasion and lymph node involvement were still absent. This finding indicates that, while still higher than in normal tissues, CALU expression gradually declines as BC advances and lymph nodes and vascular invasion occur. Afterward, the transcription level of CALU decreased as the number of affected lymph nodes increased. The tumor microenvironment (TME) may be the reason why CALU expression levels rise during the beginning of BC and fall subsequently. Even though CALU expression has decreased in cancerous tissue, it is still greater than in healthy tissues.

 To distinguish between the luminal A and luminal B stages of BC, a midpoint of 14% was proposed as the ideal limit for Ki-67 evaluation.^41^ Our analysis showed that samples with ki-67 > 14% had higher CALU mRNA expression than samples with ki-67 < 14% (Figure 2E).

 There were notable changes in CALU expression between the two groups of investigated samples based on histological grade. Tumor samples of grades 1 and 2 were included in group 1, while only grade 3 tumor samples were included in group 2. Grade 3 samples had a greater average CALU mRNA level than grades 1 and 2 samples (Figure 2F).

 It is advised to employ Histological grade^42^ and Ki-67,^43^ two significant prognostic factors in the early stages of BC, together for a more precise evaluation.^7^ Both of these characteristics and the CALU transcription were directly correlated in our study, confirming the link between the CALU and the invasive nature of BC.^5,8^

###  Evaluation of the effects of CALU knockdown on the BC cells

**Figure 3:**
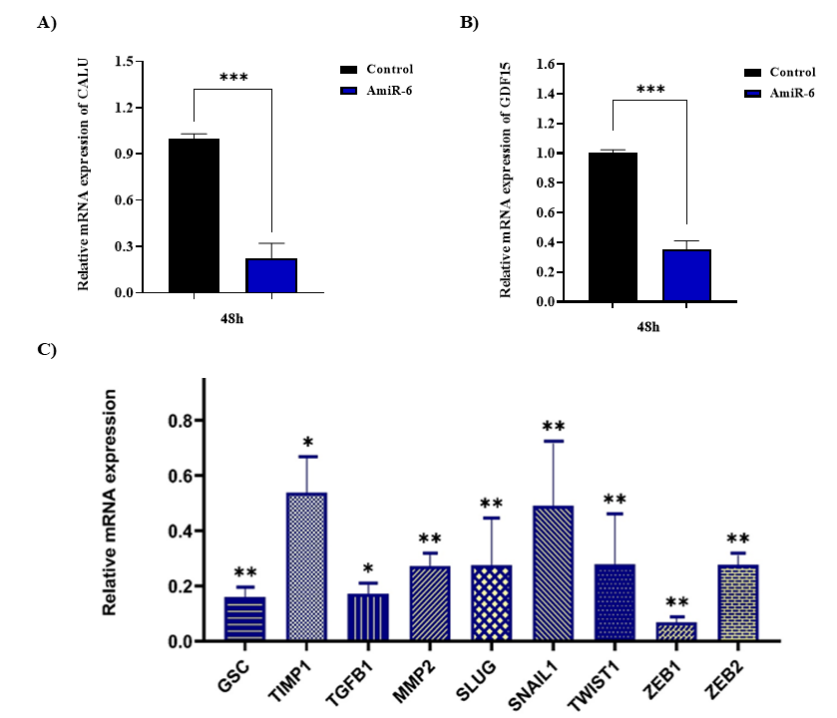
The expression levels ofCALU (A), GDF-15 (B), and EMT markers (C) in the SK-BR-3 cell line, using qRT-PCR, following the AmiR-6 overexpression, at 48h post-transfection. The names of markers are given. * = *P* ≤ 0.05, ** = *P* ≤ 0.01, *** = *P* ≤ 0.001

 When CALU transcription was evaluated in SK-BR-3 cells transfected with a plasmid encoding amiR-6, CALU was successfully knocked down (Figure 3A). GDF-15 expression was likewise reduced in these cells as a result of amiR-6-mediated CALU knockdown (Figure 3B). According to a previous study, the colorectal SW480 cell line’s GDF-15 is stimulated by the CALU nuclear isoform,CALU-15, which increases cell motility and filopodia production in colon cancer.^8^ Numerous biological processes have been reported as being regulated by GDF-15, which exhibits both tumor promoter and tumor suppressor properties.^44,45^

 GDF-15 promotes the migratory and invasive phenotype of BC cells in vitro by activating the actin cytoskeleton-regulating genes alpha Parvin (PARVA), RhoA, Rho-associated protein kinase-1 (ROCK-1), and Facsin-1, according to a systematic review conducted to revisit the potential involvement of GDF-15 in cell metastasis in various cancer types.^46^ It has previously been suggested that GDF-15 contributes to the development of BC by triggering signaling pathways that regulate EMT and cellular invasion,^47^ as well as the acquisition of characteristics similar to those of cancer stem cells.^48,49^

 While GDF-15 promotes BC cells’ migratory and invasive characteristics, its effects vary depending on the cancer type. For instance, GDF-15 has been demonstrated to have proapoptotic and anti-metastatic properties in colon carcinoma cells,^50^ lung cancer,^51^ and ovarian and prostate cancer cell lines.^52^ There is also evidence that overexpression of GDF-15 inhibits the tumorigenicity of LN-Z308 glioblastoma cell line.^53^

 Apart from its carcinogenic activity, CALU’s isoforms found in the ECM and secretory pathway were also found to have a tumor-suppressive function.^21,54^ The interaction between the extracellular CALU isoforms and fibulin-1, a member of the ECM fibulin family that is directly linked to fibronectin, is the basis for the tumor suppressor effects of the CALU isoforms.^55^ By establishing a compound with fibulin 1, the extracellular CALU isoforms stop MMP-13 from degrading it. This cooperation restricts cell migration via an integrin and extracellular signal control, as well as the protein kinases 1 and 2 (ERK1/2) signaling pathway.^21^ Through its interactions with integrins, syndecans, and cell surface receptors, Fibilin-1 mediates cell adhesion.^56-58^

###  The amiR -mediated CALU-knockdown suppressed EMT in the BC cells

 Suppression of EMT markers is anticipated to reduce the invasive and metastatic potential of BC cells in vivo, as EMT is a crucial and first stage in the metastasis cascade. To break through the basement membrane, intravasate, circulate, and extravasate at distant locations, cancer cells must undergo EMT.^59^ It has previously been hypothesized that by reversing EMT, CALU knockdown may disrupt metastasis processes and limit the ability of cancer cells to spread.^60^ To examine this hypothesis, the expression assessment of EMT-related markers, including *SNAI1*, *TGF-b1*, *ZEB1*, *ZEB2*, *SNAI2* (*SLUG*)*, GSC*, *MMP2*, *TIMP1*, and *TWIST *in the SK-BR-3 BC cell line, was performed at 48 h of post-transfection with amiR6. Overexpression of amiR-6 in the SK-BR-3 BC cell line showed inhibitory effects on the studied EMT markers (Figure 3C). This result could have been caused by GDF-15 downregulation (Figure 3B), which is corroborated by earlier studies showing that GDF-15 promotes the expression of EMT markers, SNAIL and TWIST in colorectal cancer.^48,61,62^ These two proteins play a role in suppressing the epithelial phenotype by directly inhibiting the expression of E-cadherin.^63,64^ The potential of CALU as a hallmark of EMT is supported by the results acquired thus far from the CALU knockdown technique in the SK-BR-3 cell line, which are in accord with other reports.^34,35,40,54,65^

 The regulatory role of CALU in EMT in BC was validated by Chen et al in a recent pan-cancer screening study. This investigation demonstrated that CALU, a crucial element of the TME, promotes cell migration through EMT.^26^ To further restrict invasion and metastasis, it appears that CALU knockdown may have an impact on the interactions between cancer cells and stromal cells in vivo.

 Research on gliomas has shown that EMT phenotype is closely linked to genes relevant to CALU.^5^ Several immunohistochemical tests, the Gene Expression Omnibus (GEO) database, and the Cancer Genome Atlas (TCGA) have also revealed CALU to be one of the most prevalent proteins expressed in cancer-associated fibroblasts (CAF), supporting its inclusion in the signature of EMT-related genes in gastric cancer.^66^

 According to the findings of the previous stage, which demonstrated the impact of CALU on GDF-15 expression, the results of the evaluation of the EMT marker of cells treated with amiR-6 validate the relationship between CALU and EMT markers as well as its regulatory function in cancer-related processes. The results obtained from the evaluation of the EMT marker of cells treated with amiR-6, in line with the inhibitory function of CALU on GDF-15 expression, support the association of CALU with EMT markers and cancer-related processes. While in vivo studies are needed to validate these findings, our results suggest that CALU is a promising therapeutic target for inhibiting BC metastasis.

###  Effect of amiR -mediated degradation of CALU on proliferation, apoptosis, and migration of BC cell lines

####  CALU knockdown inhibited the proliferation of BC cell lines

**Figure 4:**
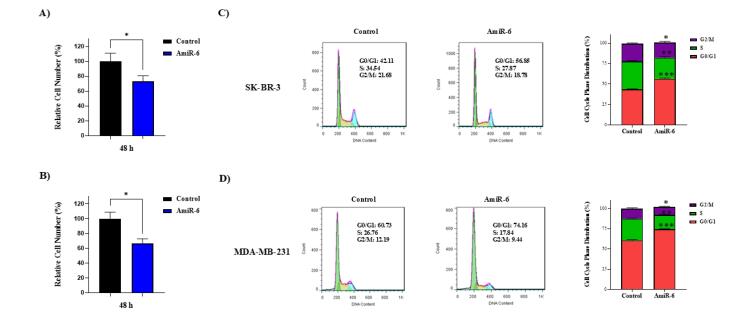
The proliferation assessment of the BC cell lines, SK-BR-3 (upper row), and MDA-MB-231 (lower row), before and after the amiR-6 overexpression. (A, B) Trypan blue staining assessments. (C, D) Cell cycle analysis. * *P* < 0.05, ** *P* < 0.01, *** *P* < 0.001)

 The SK-BR-3 cell line was assessed alongside the MDA-MB-231 cell line to examine the impact of amiR-mediated CALU knockdown on BC cell proliferation after the crucial influence of CALU on EMT was demonstrated in this cell line. Cell proliferation was then evaluated at 24 and 48 hours, using trypan blue staining techniques. At 48 hours after transfection, amiR-6 dramatically decreased cell proliferation in both treated BC cell lines, when compared to the control group (Figure 4A, B). Using cell cycle analysis, we further validated this result (Figure 4C, D). The G1 phase cell population of the amiR-6 transfected group was significantly higher than that of the control group, according to the data.

####  Knockdown of CALU-induced apoptosis in BC cell lines

 At 48 hours after amiR-6 transfection, the cell lines MDA-MB-231 and SK-BR-3 BC were simultaneously stained with ethidium bromide and acridine orange to examine the impact of CALU knockdown on apoptosis in BC cells. Acridine orange exhibits green or red fluorescences to dsDNA or ssDNA/RNA, depending on whether it is absorbed by living or dead cells. On the other hand, ethidium bromide only penetrates non-viable cells, where it attaches itself to DNA and emits red fluorescence.^67^

**Figure 5:**
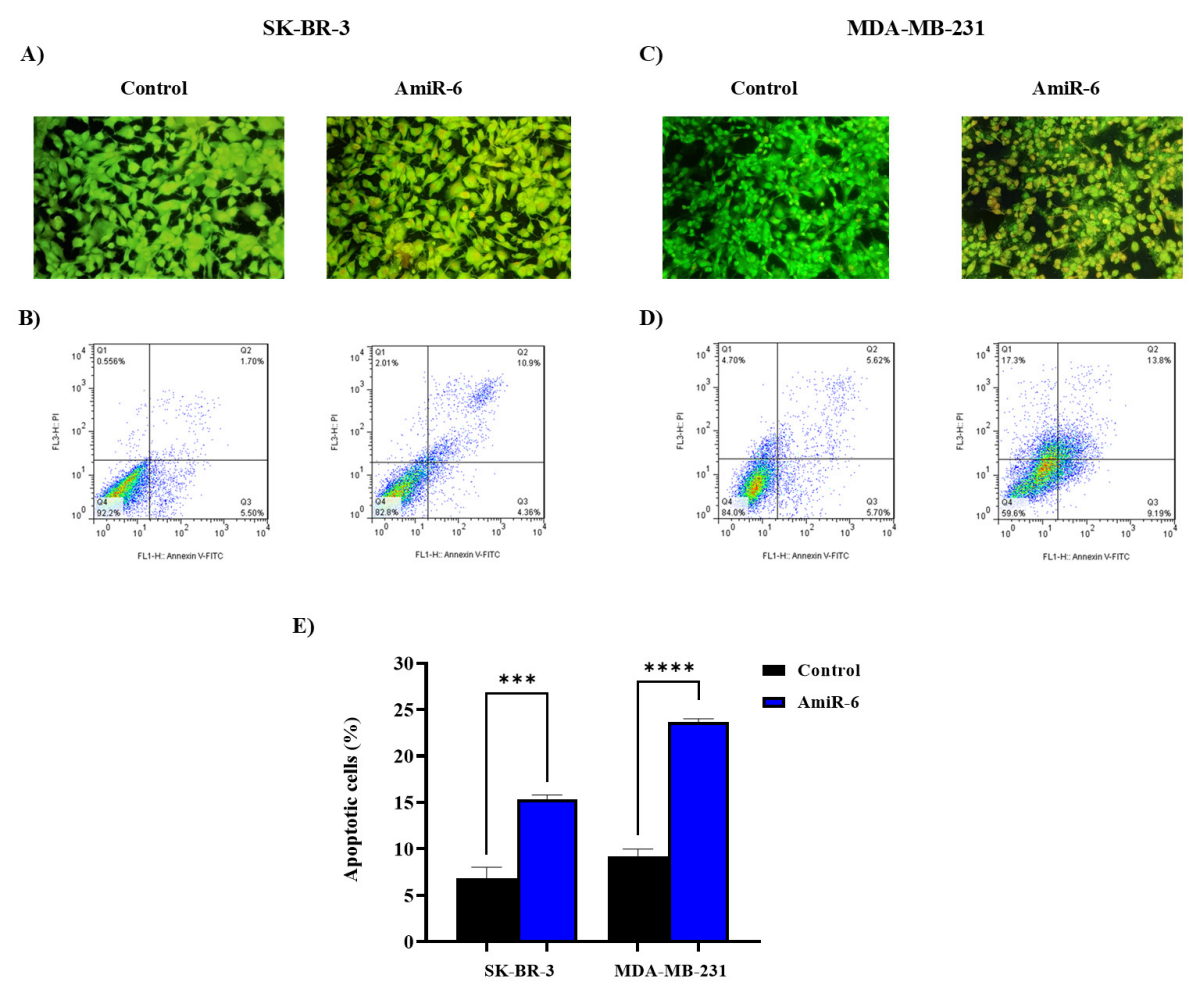
The impact of the AmiR-6 overexpression on apoptosis in the BC cell lines. Acridine orange/ethidium bromide (AO/EB) staining (A, B). Apoptosis analysis using PI staining (PI, Y-axis) and Annexin-FITC (X-axis) (C, D). (Q1 = necrotic cells, Anx- /PI + ; Q2 = late apoptotic cells, Anx + /PI + ; Q3 = early apoptotic cells, Anx + /PI-; and Q4 = living cells, Anx-/ PI-). E) “Quantification of apoptosis (%) in SK-BR-3, and MDA-MB-231cells after CALU knockdown”

 The cells transfected with amiR-6 were yellow to orange, suggesting the presence of apoptosis, whereas both BC cell lines analyzed in the control group were green, showing the lack of apoptosis (Figure 5A, C). The apoptotic cells were confirmed and measured by flow cytometry analysis. Approximately 15.44% of the transfected SK-BR-3 cells accounted for apoptotis (early and late apoptosis) (Figure 5B), while 23.21% of the transfected MD-MB-231 cells accounted for apoptotis (Figure 5D). Estimation of the percentage of apoptosis in both SK-BR-3 and MDA-MB-231 cells after transfection with amiR-6, suggests that the CALU-knockdown significantly increased apoptosis in the BC cells (Figure 5E).

####  Knockdown of CALU inhibited the migration of BC cell lines

**Figure 6:**
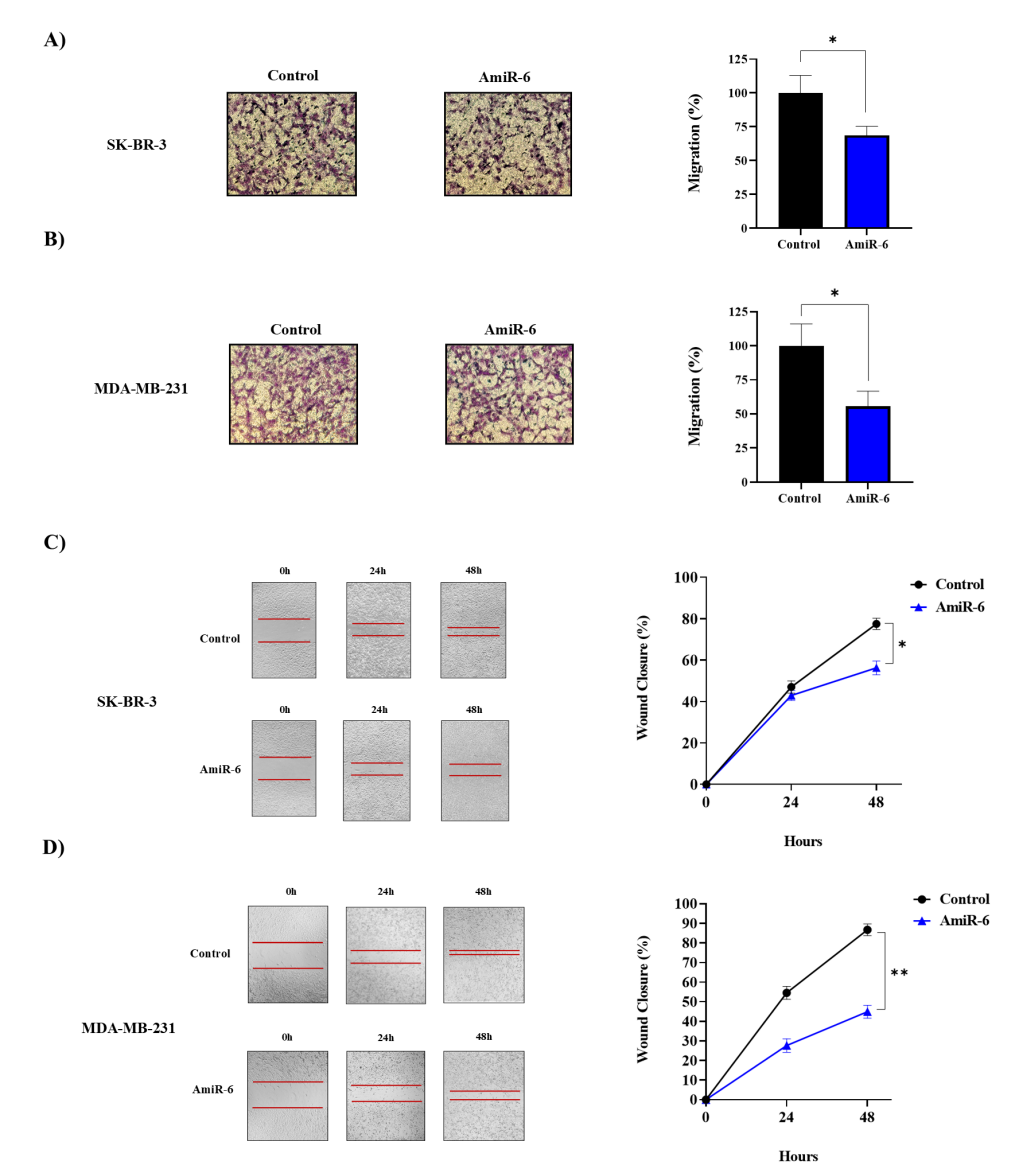
The effect of the amiR6-mediated CALU knockdown on the migration rates of the BC cell lines, by performing wound healing assay. The microscopic picture of the cells (A), and the migration rate (%) assessment of the cells at 0, 24, and, 48 h of post-transfection (B). The distances of the cell-free surface, are measured by image j software (C, D). * = *P* ≤ 0.05, ** = *P* ≤ 0.01.

 A transwell experiment was used to investigate the impact of amiR-6-mediated CALU-knockdown on the migration of two BC cell lines. The results demonstrated a significant decrease in the migratory capacity of both cell lines 48 hours after transfection (Figure 6). According to our results, fewer migratory cells adhered to the bottom of the chamber when CALU was knocked down by amiR-6 (Figure 6A, B). In addition to the transwell migration test, a wound healing experiment was used to show how CALU knockdown affected the migration of BC cells. The migratory rate of the BC cells in the scratched areas decreased significantly for both BC cell lines after amiR-6 expression (Figure 6C, D). The conclusion, that CALU can successfully affect BC cell migration, was corroborated by the results of the transwell migration and wound healing assays.

 After demonstrating the beneficial effect of CALU on EMT in SKBR3, we demonstrated that CALU knockdown results in cell cycle arrest, a higher percentage of necrotic and apoptotic cells, and decreased proliferation and migration in both BC cell lines by using the knockdown technique in both SKBR3 and MDA-MB-231 cell lines. These findings are consistent with the EMT marker analysis results, demonstrating the critical function of CALU in BC metastatic promotion. In colon and lung tumors, our group recently described the involvement of CALU in inducing metastases.^22^ These results are in line with reports that indicate CALU plays a role in increasing the invasion, migration, metastasis, and proliferation of cancer cells during the carcinogenesis process.^68,69^ In more recent work, Li and colleagues reported the promoting function of CALU in lung adenocarcinoma progression by enhancing cell proliferation, migration, and invasion.^40^ Consistent with these findings, upregulation of CALU and other known CAF markers, such as periostin (POSTN, or PSTN), α-smooth muscle actin, and podoplanin, was demonstrated in lung adenocarcinoma, suggesting CALU, as a CAF-derived molecule, act effectively in cancer promotion.^9^ Focusing on TNBC-specific mechanisms, that allow the expansion and activity of self-renewing therapy-resistant cancer stem cells (CSC), CALU was found among the Top 30 upregulated genes in CD44-high/CD24-low cluster and suggested as a CSC regulator in TNBC and other cancers.^70^ Thus, strategies aimed at inhibiting CALU expression or activity could potentially disrupt cancer progression, sensitize cancer cells to apoptosis, inhibit EMT, and reduce metastasis.^71^

 CALU regulates numerous cellular functions in significant ways.^72,73^ It promotes vascular calcification by reducing gamma-carboxylation and inhibiting the activation of matrix Gla protein.^57,58^ Moreover, CALU is crucial for maintaining calcium homeostasis,^1^ and controlling the uptake of Ca2 + during smooth muscle and cardiac contraction and excitation.^74^ Furthermore, CALU plays a part in iron-dependent cell death, gene alterations, and TME remodeling.^6^ It has been proposed that CALU is an upstream regulator that affects several pathways and, consequently, the development of cancer.^40^ Both in vitro and in vivo, the amiR-mediated CALU knockdown of mucosal melanoma cells caused apoptosis and cell cycle arrest while suppressing cell growth, migration, invasion, and metastasis, most likely by blocking phosphorylated extracellular signal-regulated kinase (ERK) signaling.^69^ Additionally, it has been demonstrated that CALU regulates apoptosis, and that apoptosis dysregulation leads to cancer cell survival and chemotherapy resistance.^68,75,76^

 The CALU knockdown’s impact on cell migration may be mediated through disruptions in key signaling pathways such as integrin, MAPK, and PI3K/AKT. Integrins, essential for cell-ECM interactions and migration, could be affected by CALU’s role in EMT and TME modulation.^77,78^ Furthermore, CALU may influence the MAPK pathway via GDF-15,^79,80^ and GDF-15 can activate AKT, impacting downstream targets involved in cell survival, growth, and migration.^81,82^ However, further investigation is required to fully elucidate the specific mechanisms and confirm the involvement of these pathways in CALU’s regulation of BC cell migration.

 In tumor tissues from mice with BC, Menon, and colleagues found that the CALU-1 isoform was up-regulated whereas CALU-2 expression remained unchanged,^83^ supporting the idea that CALU isoforms are differentially expressed. In this context, it is noteworthy that CALU-15 has been shown to have the opposite effect on tumor progression when compared to other CALU isoforms of the secretory pathway.^8,21^ This finding may contribute to the regulating role of CALU in processes connected to cancer. Given the proposed function of CALU-15, more research is necessary to clarify the mechanisms of action of CALU isoforms and their potential connection to processes connected to BC.

 The CALU-mediated knockdown approach with CALU-specific miRNA adopted in this study revealed the impact of CALU on the gene(s) or pathways associated with BC progression. However, when it comes to cancer treatment, this approach could also provide a suitable strategy for targeted therapy approaches by silencing gene(s) that are involved in tumorigenesis. The miRNA-mediated knockdown holds promise, because of its ability to modulate gene expression levels, without turning it off, impacting key pathways involved in cancer progression. The clinical implications of this approach are vast, from direct tumor suppression to combat therapeutic resistance, even in personalized cancer therapies, and in combination cancer therapies.^23,84,85^

 Our study has limitations, including its in vitro nature, limited cell lines, potential off-target microRNA effects, unclear molecular mechanisms, and small clinical sample size. These factors should be considered when interpreting the results. Future research should involve in vivo studies, more diverse cell lines, stricter microRNA controls, mechanistic investigations, larger clinical cohorts, and blind reviewing of clinicopathological features.

## Conclusion

 Altogether, the evidence presented in this study, including the upregulation of CALU in BC tissues and its correlation with the main clinicopathological features in BC patients, in addition to the result of CALU knockdown, have confirmed the metastasis-promoting role of CALU, which occurs via inhibiting apoptosis and enhancing the proliferation, migratory and invasive abilities of BC cells. These intriguing observations warrant further investigation into the specific mechanisms of CALU’s regulatory role. Understanding these mechanisms and their association with different CALU isoforms could lead to the development of CALU-targeted therapeutic interventions tailored to specific types of cancer, thus offering a promising path for clinical translation. In addition, our study provides valuable experimental and foundational data for further research and a more comprehensive understanding of the molecular function of CALU in BC.

 A key strength is our use of a amiR-mediated knockdown approach, which may offer advantages in terms of long-term gene silencing compared to traditional siRNA methods. Furthermore, our study provides strong evidence for a correlation between CALU expression and a comprehensive set of clinicopathological features in BC patients. We also performed a detailed analysis of the effects of CALU knockdown on various cellular processes. However, like many in vitro studies, our findings may not fully recapitulate the complexity of the TME in vivo. In addition, the precise molecular mechanisms underlying the effects of CALU knockdown remain unclear, and our clinical sample size was relatively small. Future studies may address these limitations.

## Competing Interests

 None

## Data Availability Statement

 The data that supports the findings of this study are available in the supporting materials of this article. Further detailed experimental data that support the findings of this study are available from the corresponding author upon reasonable request.

## Patient Consent Statement

 Concerning the tissue samples, used in this work, written informed consent was obtained for all cases by clinicians.
